# Supplementary material for: T cells in testicular germ cell tumors: new evidence of fundamental contributions by rare subsets
Source: Br J Cancer. 2024 Apr 22;130(12):1893–903. doi: 10.1038/s41416-024-02669-9 (PMC11183042; doi:10.1038/s41416-024-02669-9)
Supplement: Supplementary file 1 — Supplementary information [file 41416_2024_2669_MOESM1_ESM.pdf]

Supplemental Table 1: List of antibodies used for IHC and their working conditions.

**Primary antibody**	**Specificty**	**Supplier and Catalogue no**	**Dilution**	**Staining**
Polyclonal rabbit anti-human CD3	Pan T cells	DAKO, A0452	1:100	AEC
Monoclonal mouse anti-human CD20cy	B cells	DAKO, M0755	1:100	AEC
Monoclonal mouse anti-human CD68	Macrophages	DAKO, M0876	1:100	NovaRED
Monoclonal mouse anti-human CD11c	Dendritic cells	Novocastra, NCL-L-CD11c-563	1:100	NovaRED
Monoclonal rabbit anti-human CD4	T helper (Th) cells	Abcam, ab133616	1:100	AEC
Monoclonal mouse anti-human CD8a	Cytotoxic T (Tc) cells	eBioscience, Product: 14-0008-82	1:250	AEC
Polyclonal rabbit anti-human IL2RA (CD25)	Treg	Sigma, HPA054622	1:500	AEC
Monoclonal mouse anti-human FOXP3	Treg	eBioscience, Product: 14-4777-80	1:100	AEC
Polyclonal rabbit anti-human CXCR5	Tfh	Sigma, HPA042432	1:2000	AEC
Monoclonal mouse anti-human BCL6	Tfh	DAKO, M7211	1:50	AEC
**Secondary antibody**				
Biotinylated goat anti-rabbit		DAKO, E0432	1:100	
Biotinylated goat anti-mouse		DAKO, E0433	1:100	

Supplemental Table 2: Statistical analysis of infiltration density and spatial distribution of different immune cell types identified by IHC in human testis specimens: multiple comparison across the different sample categories. Statistical significance was tested by a non-parametric Kruskal-Wallis-H test including Dunn's multiple comparisons test (p-values ≥ 0.05 were considered as not significant).

			NSP (n=10)	HYP+ly (n=11)	GCNIS (n=14)	GCNIS+ly (n=12)	SE (n=24)	EC (n=10)
CD3	**Median infiltration density**		1.50	3.00	2.00	3.50	4.00	4.00
	Adjusted P value	vs. NSP		0.1400	>0.9999	**0.0052**	**<0.0001**	**<0.0001**
		vs. HYP+LY			>0.9999	>0.9999	**0.0286**	0.3463
		vs. GCNIS				0.3914	**<0.0001**	**0.0037**
		vs. GCNIS+LY					0.1633	>0.9999
		vs. SE						>0.9999)
	Distribution (%)	Disseminated	80.00	27.00	64.00	19.00	87.50	50.00
		Multifocal		55.00	29.00	75.00	12.50	40.00
		Focal	10.00	18.00	7.00	6.00		10.00
		Absent	10.00					
CD4	**Median infiltration density**		2.00	3.00	3.00	4.00	4.00	3.50
	Adjusted P value	vs. NSP		**0.0087**	0.8535	**0.0004**	**<0.0001**	**0.0059**
		vs. HYP+LY			>0.9999	>0.9999	>0.9999	>0.9999
		vs. GCNIS				0.1789	**0.0037**	0.6624
		vs. GCNIS+LY					>0.9999	>0.9999
		vs. SE						>0.9999
	Distribution (%)	Disseminated	90.00	33.00	79.00	40.00	88.00	50.00
		Multifocal		42.00	21.00	60.00	8.00	50.00
		Focal		25.00			4.00	
		Absent	10.00					
CD8	**Median infiltration density**		0.75	3.00	2.00	3.00	3.50	3.50
	Adjusted P value	vs. NSP		0.0822	>0.9999	**0.0011**	**<0.0001**	**<0.0001**
		vs. HYP+LY			>0.9999	>0.9999	0.2362	0.6192
		vs. GCNIS				0.3570	**0.0016**	**0.0283**
		vs. GCNIS+LY					>0.9999	>0.9999
		vs. SE						>0.9999
	Distribution (%)	Disseminated	70	9.00	71.00	13.00	100.00	40.00
		Multifocal		64.00	29.00	80.00		50.00
		Focal	30	27.00		7.00		10.00
		Absent						
CD20cy	**Median infiltration density**		0.00	1.00	0.00	0.25	2.50	2.00
	Adjusted P value	vs. NSP		0.2827	>0.9999	0.7568	**<0.0001**	**0.0097**
		vs. HYP+LY			0.9534	>0.9999	**0.0338**	>0.9999
		vs. GCNIS				>0.9999	**<0.0001**	**0.0372**
		vs. GCNIS+LY					**0.0024**	>0.9999
		vs. SE						>0.9999
	Distribution (%)	Disseminated	100.00		7.00		36.00	55.00
		Multifocal		7.00		19.00	57.00	36.00
		Focal		50.00	14.00	31.00	3.50	9.00
		Absent		43.00	79.00	50.00	3.50	
CD68	**Median infiltration density**		2.75	2.50	2.00	2.50	3.50	3.00
	Adjusted P value	vs. NSP		>0.9999	>0.9999	>0.9999	**0.0477**	>0.9999
		vs. HYP+LY			>0.9999	>0.9999	**0.0035**	0.5186
		vs. GCNIS				>0.9999	**<0.0001**	0.0592
		vs. GCNIS+LY					**0.0007**	0.2497
		vs. SE						>0.9999
	Distribution (%)	Disseminated	100.00	64.00	93.00	87.00	92.50	73.00
		Multifocal		14.50		10.00	7.50	27.00
		Focal		21.50				
		Absent			7.00			

**CD11c**	**Median infiltration density**		0.00	0.50	1.00	2.50	3.50	4.00
	Adjusted P value	vs. NSP		>0.9999	>0.9999	>0.9999	**<0.0001**	**<0.0001**
		vs. HYP+LY			>0.9999	>0.9999	**<0.0001**	**0.0006**
		vs. GCNIS				>0.9999	**<0.0001**	**0.0003**
		vs. GCNIS+LY					**0.0008**	**0.0057**
		vs. SE						>0.9999
	Distribution (%)	Disseminated	10.00		53.00	13.00	75.00	30.00
		Multifocal		33.00	7.00	33.00	25.00	50.00
		Focal	30.00	17.00	13.00	27.00		10.00
		Absent	60.00	50.00	27.00	27.00		10.00

Supplemental Table 3 : List of the antibodies with their conjugated dye used for Flow cytometric analysis and their working conditions. All antibodies were purchased from Miltenyi Biotec (except FOXP3, Biolegend). Intercellular targeted antibodies are highlighted in blue.

**Channel**	**PANEL 1**		**Channel**	**PANEL 2**	
**V1**	CD3-VioBlue, Clone: REA613, Cat. 130-114-710	1:50	**V1**	CD3-VioBlue, Clone: REA613, Cat. 130-114-519	1:50
**V2**	Viobility™ 405/520 Fixable Dye, Cat. 130-109-814	1µl	**V2**	Viobility™ 405/520 Fixable Dye, Cat. 130-109-814	1µl
**B1**	CD45-VioBright 515, Clone: REA747, Cat. 130-110-640	1:50	**B1**	CD25-VioBright 515, Clone: REA570, Cat. 130-113-287	1:50
**B2**	CD20cy-PE, Clone: REA543, Cat. 130-108-313	1:11	**B2**	BCL6-PE, Clone: REA373, Cat. 130-118-346	1:50
**B3**	CD4-PerCP-Vio700, Clone: REA623, Cat. 130-113-790	1:50	**B3**	CD4-PerCP-Vio700, Clone: REA623, Cat. 130-113-228	1:50
**B4**	CD8-PE-Vio770, Clone: REA734, Cat. 130-110-818	1:50	**B4**	CD185 (CXCR5)-PE-Vio770, Clone: REA103, Cat. 130-117-508	1:50
**R1**	Alexa Fluor® 647 anti-human FOXP3 Antibody, Clone: 206D , Cat: 320114, Biolegend	1:50	**R1**	Alexa Fluor® 647 anti-human FOXP3 Antibody, Clone: 206D, Cat: 320114, Biolegend	1:50
**R2**	CD68-APC-Vio770, Clone: REA886, Cat. 130-114-463	1:50	**R2**	CD45-APC-Vio770, Clone: REA747, Cat. 130-110-635	1:50

Supplemental Table 4: Statistical analysis of different cells of the multiple comparisons across the different localisation of TGCT samples after performing flow cytometry. The mean of each column was compared with the mean of every other column. Significance tested by ordinary one-way ANOVA including Tukey's Honest Significant Difference Test p-values ≥ 0.05 considered as not significant (for better assessment, statistically significant comparisons are written in bold letters and highlighted).

	**CD3+ cells**	**CD4+ cells**	**CD8+ cells**	**CD68+ cells**	**CD20cy+ cells**	**CD25+FOXP3+ cells**	**CXCR5+BCL6+ cells**
Seminoma-Tumor vs. Seminoma-Tumor-Adj	**0.0012**	**0.0003**	0.3704	>0.9999	0.9974	**0.0374**	0.2294
Seminoma-Tumor vs. Seminoma-Tumor-Dis	**<0.0001**	**<0.0001**	0.0512	0.9786	>0.9999	0.4000	0.9782
Seminoma-Tumor vs. Seminoma-Contralateral 1	**<0.0001**	**<0.0001**	0.0525	>0.9999	0.9859	**0.0380**	0.8401
Seminoma-Tumor vs. Seminoma-Contralateral 2	**<0.0001**	**<0.0001**	**0.0173**	>0.9999	0.9227	**0.0022**	0.2904
Seminoma-Tumor vs. Embryonal carcinoma (≥80%) -Tumor	0.9896	>0.9999	>0.9999	0.0946	0.9989	0.5582	0.7885
Seminoma-Tumor vs. Mixed Tumors-Tumor	0.1883	0.9767	0.9469	0.5506	0.9254	0.1002	0.9639
Seminoma-Tumor-Adj vs. Seminoma-Tumor-Dis	0.9985	0.8453	0.9998	0.9774	>0.9999	>0.9999	0.9978
Seminoma-Tumor-Adj vs. Seminoma-Contralateral 1	0.9645	0.9780	0.9901	>0.9999	>0.9999	>0.9999	>0.9999
Seminoma-Tumor-Adj vs. Seminoma-Contralateral 2	0.7251	0.9256	0.9609	>0.9999	>0.9999	0.9992	>0.9999
Seminoma-Tumor-Adj vs. Embryonal carcinoma (≥80%)-Tumor-Adj	0.8698	0.9776	>0.9999	0.9949	>0.9999	0.9998	>0.9999
Seminoma-Tumor-Adj vs. Mixed Tumors-Tumor-Adj	0.9993	>0.9999	>0.9999	>0.9999	>0.9999	>0.9999	>0.9999
Seminoma-Tumor-Dis vs. Seminoma-Contralateral 1	>0.9999	>0.9999	>0.9999	0.9998	>0.9999	0.9997	>0.9999
Seminoma-Tumor-Dis vs. Seminoma-Contralateral 2	0.9986	>0.9999	>0.9999	0.99939	0.9996	0.9431	0.9985
Seminoma-Tumor-Dis vs. Embryonal carcinoma (≥80%)-Tumor-Dis	0.9998	>0.9999	>0.9999	0.9987	0.9999	0.9954	>0.9999
Seminoma-Tumor-Dis vs. Mixed Tumors-Tumor-Dis	>0.9999	>0.9999	>0.9999	>0.9999	>0.9999	>0.9999	>0.9999
Seminoma-Contralateral 1 vs. Seminoma-Contralateral 2	>0.9999	>0.9999	>0.9999	>0.9999	>0.9999	>0.9999	>0.9999
Seminoma-Contralateral 1 vs. Embryonal carcinoma (≥80%)-Contralateral 1	>0.9999	>0.9999	>0.9999	0.8979	>0.9999	>0.9999	>0.9999
Seminoma-Contralateral 1 vs. Mixed Tumors-Contralateral 1	>0.9999	>0.9999	>0.9999	>0.9999	>0.9999	>0.9999	0.9994

Seminoma-Contralateral 2 vs. Embryonal carcinoma (≥80%)-Contralateral 2	>0.9999	>0.9999	0.7568	>0.9999	>0.9999	>0.9999	>0.9999
Seminoma-Contralateral 2 vs. Mixed Tumors-Contralateral 2	>0.9999	>0.9999	>0.9999	0.8244	>0.9999	>0.9999	>0.9999
Embryonal carcinoma (≥80%) -Tumor vs. Embryonal carcinoma (≥80%)-Tumor-Adj	**0.0180**	**0.0052**	0.2172	0.9493	>0.9999	0.9813	>0.9999
Embryonal carcinoma (≥80%) -Tumor vs. Embryonal carcinoma (≥80%)-Tumor-Dis	**0.0281**	**0.0018**	0.7592	0.4189	>0.9999	0.9958	>0.9999
Embryonal carcinoma (≥80%) -Tumor vs. Embryonal carcinoma (≥80%)-Contralateral 1	**0.0104**	**0.0017**	0.0713	>0.9999	>0.9999	0.9974	>0.9999
Embryonal carcinoma (≥80%) -Tumor vs. Embryonal carcinoma (≥80%)-Contralateral 2	**0.0126**	**0.0216**	0.9910	0.8643	>0.9999	0.9985	>0.9999
Embryonal carcinoma (≥80%) -Tumor vs. Mixed Tumors-Tumor	0.9838	>0.9999	0.8718	>0.9999	0.5186	>0.9999	>0.9999
Embryonal carcinoma (≥80%)-Tumor-Adj vs. Embryonal carcinoma (≥80%)-Tumor-Dis	>0.9999	>0.9999	>0.9999	0.9995	>0.9999	>0.9999	>0.9999
Embryonal carcinoma (≥80%)-Tumor-Adj vs. Embryonal carcinoma (≥80%)-Contralateral 1	>0.9999	>0.9999	>0.9999	0.9994	>0.9999	>0.9999	>0.9999
Embryonal carcinoma (≥80%)-Tumor-Adj vs. Embryonal carcinoma (≥80%)-Contralateral 2	>0.9999	>0.9999	0.9892	>0.9999	>0.9999	>0.9999	>0.9999
Embryonal carcinoma (≥80%)-Tumor-Adj vs. Mixed Tumors-Tumor-Adj	>0.9999	0.9966	>0.9999	>0.9999	>0.9999	0.9949	>0.9999
Embryonal carcinoma (≥80%)-Tumor-Dis vs. Embryonal carcinoma (≥80%)-Contralateral 1	>0.9999	>0.9999	0.9964	0.8427	>0.9999	>0.9999	>0.9999
Embryonal carcinoma (≥80%)-Tumor-Dis vs. Embryonal carcinoma (≥80%)-Contralateral 2	>0.9999	>0.9999	>0.9999	>0.9999	>0.9999	>0.9999	>0.9999
Embryonal carcinoma (≥80%)-Tumor-Dis vs. Mixed Tumors-Tumor-Dis	>0.9999	>0.9999	0.9998	>0.9999	>0.9999	0.9996	>0.9999
Embryonal carcinoma (≥80%)-Contralateral 1 vs. Embryonal carcinoma (≥80%)-Contralateral 2	>0.9999	>0.9999	0.8593	0.9936	>0.9999	>0.9999	>0.9999
Embryonal carcinoma (≥80%)-Contralateral 1 vs. Mixed Tumors-Contralateral 1	>0.9999	>0.9999	>0.9999	0.9848	>0.9999	>0.9999	>0.9999
Embryonal carcinoma (≥80%)-Contralateral 2 vs. Mixed Tumors-Contralateral 2	>0.9999	>0.9999	0.8280	0.9983	>0.9999	>0.9999	>0.9999
Mixed Tumors-Tumor vs. Mixed Tumors-Tumor-Adj	0.8917	0.4101	0.9927	0.9893	0.3646	>0.9999	0.9991
Mixed Tumors-Tumor vs. Mixed Tumors-Tumor-Dis	0.4644	**0.0296**	0.9780	0.9720	0.3468	>0.9999	>0.9999
Mixed Tumors-Tumor vs. Mixed Tumors-Contralateral 1	0.2494	**0.0262**	0.8948	0.9926	0.3842	>0.9999	0.9904

Mixed Tumors-Tumor vs. Mixed Tumors-Contralateral 2	0.2989	0.1501	0.8933	>0.9999	0.6255	0.9973	0.9975
Mixed Tumors-Tumor-Adj vs. Mixed Tumors-Tumor-Dis	>0.9999	0.9982	>0.9999	>0.9999	>0.9999	>0.9999	>0.9999
Mixed Tumors-Tumor-Adj vs. Mixed Tumors-Contralateral 1	0.9955	0.9810	>0.9999	>0.9999	>0.9999	>0.9999	>0.9999
Mixed Tumors-Tumor-Adj vs. Mixed Tumors-Contralateral 2	0.9980	>0.9999	>0.9999	0.9936	>0.9999	0.9646	>0.9999
Mixed Tumors-Tumor-Dis vs. Mixed Tumors-Contralateral 1	>0.9999	>0.9999	>0.9999	>0.9999	>0.9999	>0.9999	>0.9999
Mixed Tumors-Tumor-Dis vs. Mixed Tumors-Contralateral 2	>0.9999	>0.9999	>0.9999	0.9834	>0.9999	0.9718	>0.9999
Mixed Tumors-Contralateral 1 vs. Mixed Tumors-Contralateral 2	>0.9999	>0.9999	>0.9999	0.9949	>0.9999	0.9995	>0.9999

Supplemental Table 5: Number of cells in the scRNA-seq data sets analyzed in the current study.

<table>
<tr><th></th><th></th><th colspan="6">Cell number</th></tr>
<tr><td></td><td></td><td>Estimated cell number (cell ranger 10x)</td><td>Primary aggregation (only Donors)</td><td>Secondary aggregation (all samples)</td><td colspan="3">Cell after filtration and normalization</td></tr>
<tr><td rowspan="2">Donor1</td><td>Technical replicate 1</td><td>4276</td><td rowspan="2">7628</td><td rowspan="10">44012</td><td rowspan="2">1592</td><td rowspan="6">4440</td><td rowspan="10">10153</td></tr>
<tr><td>Technical replicate 2</td><td>3352</td></tr>
<tr><td rowspan="2">Donor2</td><td>Technical replicate 1</td><td>3744</td><td rowspan="2">7627</td><td rowspan="2">2748</td></tr>
<tr><td>Technical replicate 2</td><td>3883</td></tr>
<tr><td rowspan="2">Donor3</td><td>Technical replicate 1</td><td>4402</td><td rowspan="2">10033</td><td rowspan="2">2773</td></tr>
<tr><td>Technical replicate 2</td><td>5631</td></tr>
<tr><td>Tumor12MIX</td><td>-</td><td>5878</td><td>-</td><td>2327</td><td rowspan="4">5713</td></tr>
<tr><td>Tumor13SE</td><td>-</td><td>6397</td><td>-</td><td>2440</td></tr>
<tr><td>Tumor2EC</td><td>-</td><td>347</td><td>-</td><td>208</td></tr>
<tr><td>Tumor4SE</td><td>-</td><td>6102</td><td>-</td><td>738</td></tr>
</table>

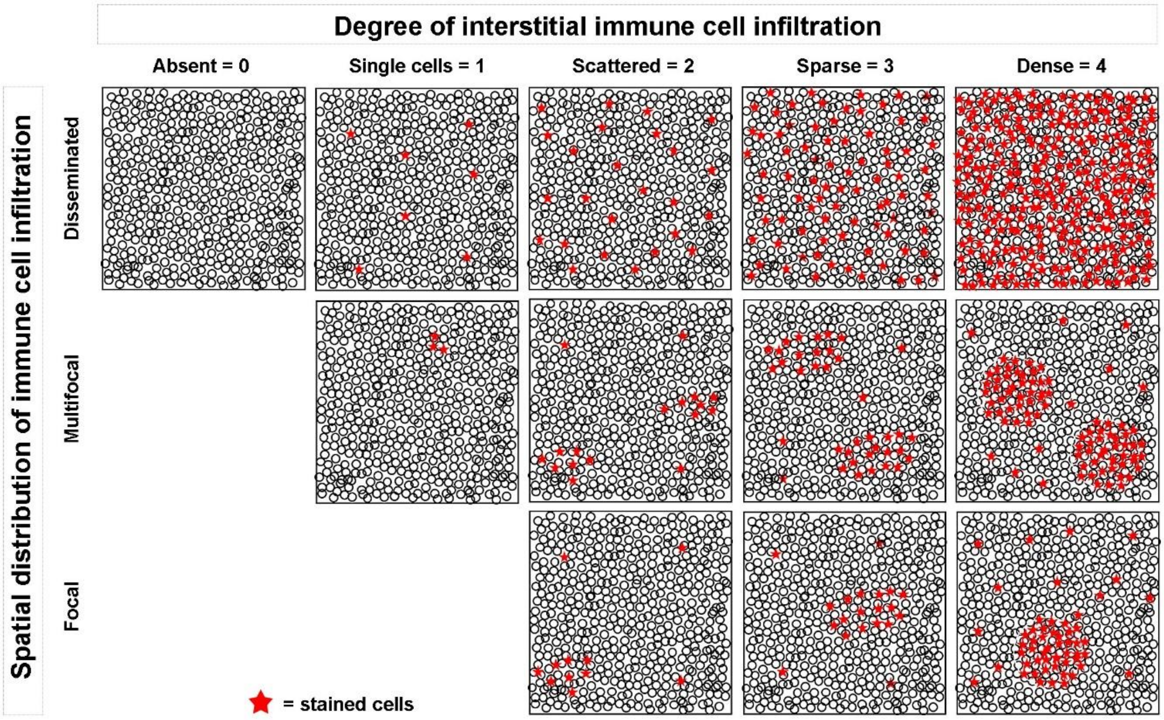

Supplemental Fig. 1: Schematic presentation of the semi-quantitative scoring system to describe the degree and distribution of immune cell infiltration. The X-axis shows the infiltration density (absent = 0, single cells = 1, scattered = 2, sparse = 3, dense = 4) and the Y-axis shows the spatial distribution patterns (focal, multifocal, and disseminated).

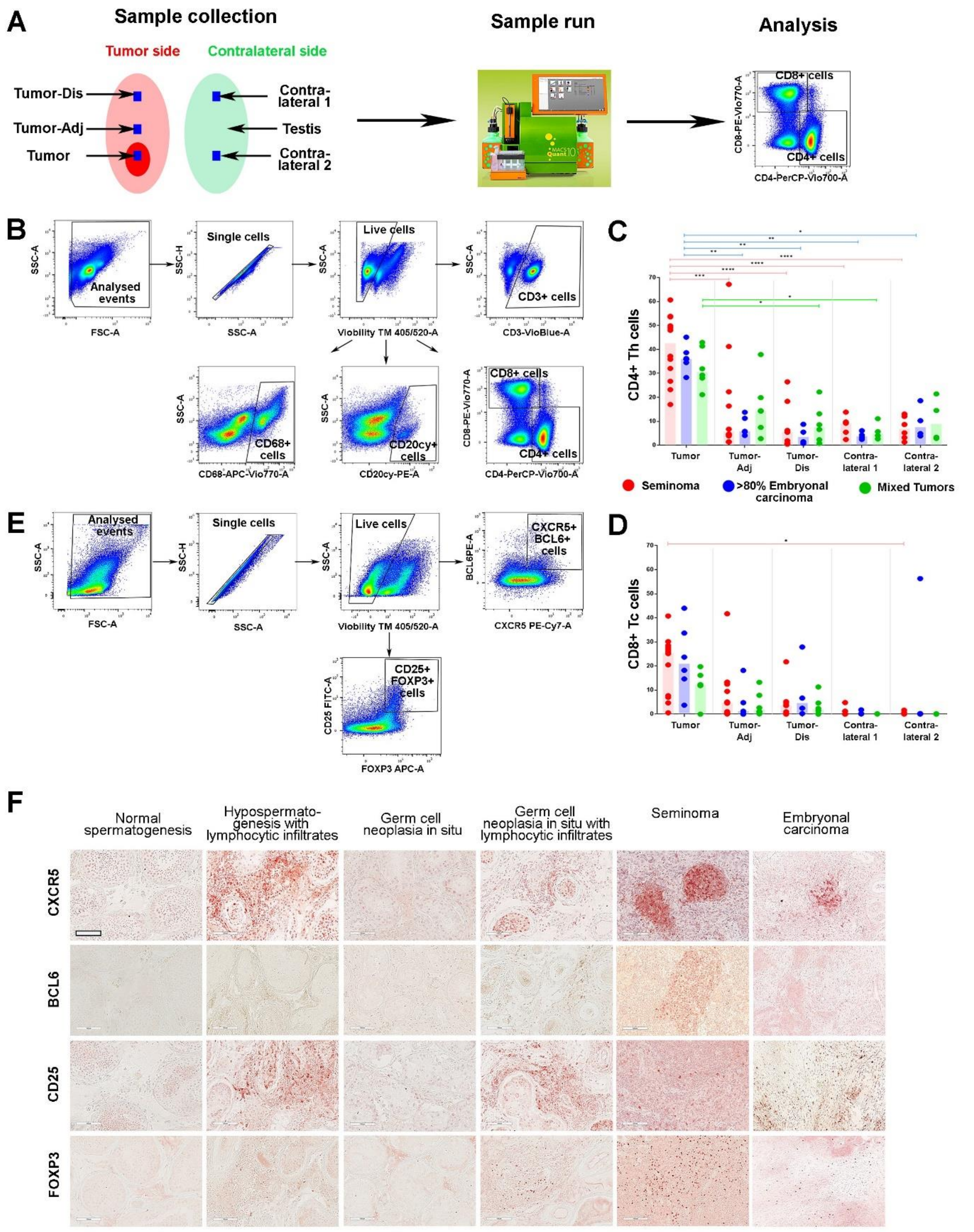

Supplemental Fig. 2: Analysis of immune cells of human testis samples by flow cytometry and IHC. A: Schematic illustration of sample collection sites during surgery of TGCT (Tumor: tumor central; Tumor-Adj: adjacent to tumor; Tumor-Dis: distant from tumor; Contralateral 1: upper pole of the contralateral testis, Contralateral 2: lower pole of the contralateral testis), and workflow of flow cytometry. **B:** Flow cytometry using antibody panel-1 (CD45, CD20cy, CD68, CD3, CD4, CD8) and respective gating

strategy to analyze different testicular immune cells. **C-D:** presence of CD4+ and CD8+ T cells in total live cells,; CD4+ T cells are more frequent in “Tumor” areas of seminoma than in other localizations and compared to CD8+ T cells (columns indicate median values for each subgroup; significance tested by ordinary one-way ANOVA including Tukey’s Honest Significant Difference Test; *p<0.05, **p<0.01, ***p<0.001; see supplemental table 4). **E:** Antibody panel-2 (CD45, CD3, CD4, CD25, FOXP3, CXCR5, BCL6) and respective gating strategy to analyze Treg and Tfh cells. **F:** Corresponding results of IHC analysis (seminoma, n=26 and embryonal carcinoma, n=10) compared to NSP (n=10), HYP+LY (n=12), GCNIS (n=14), GCNIS+LY (n=15) showing that Treg and Tfh cells are mostly found in TGCT samples, especially in seminoma. All images at the same magnification, white bar in top left-hand panel indicates 100µm.

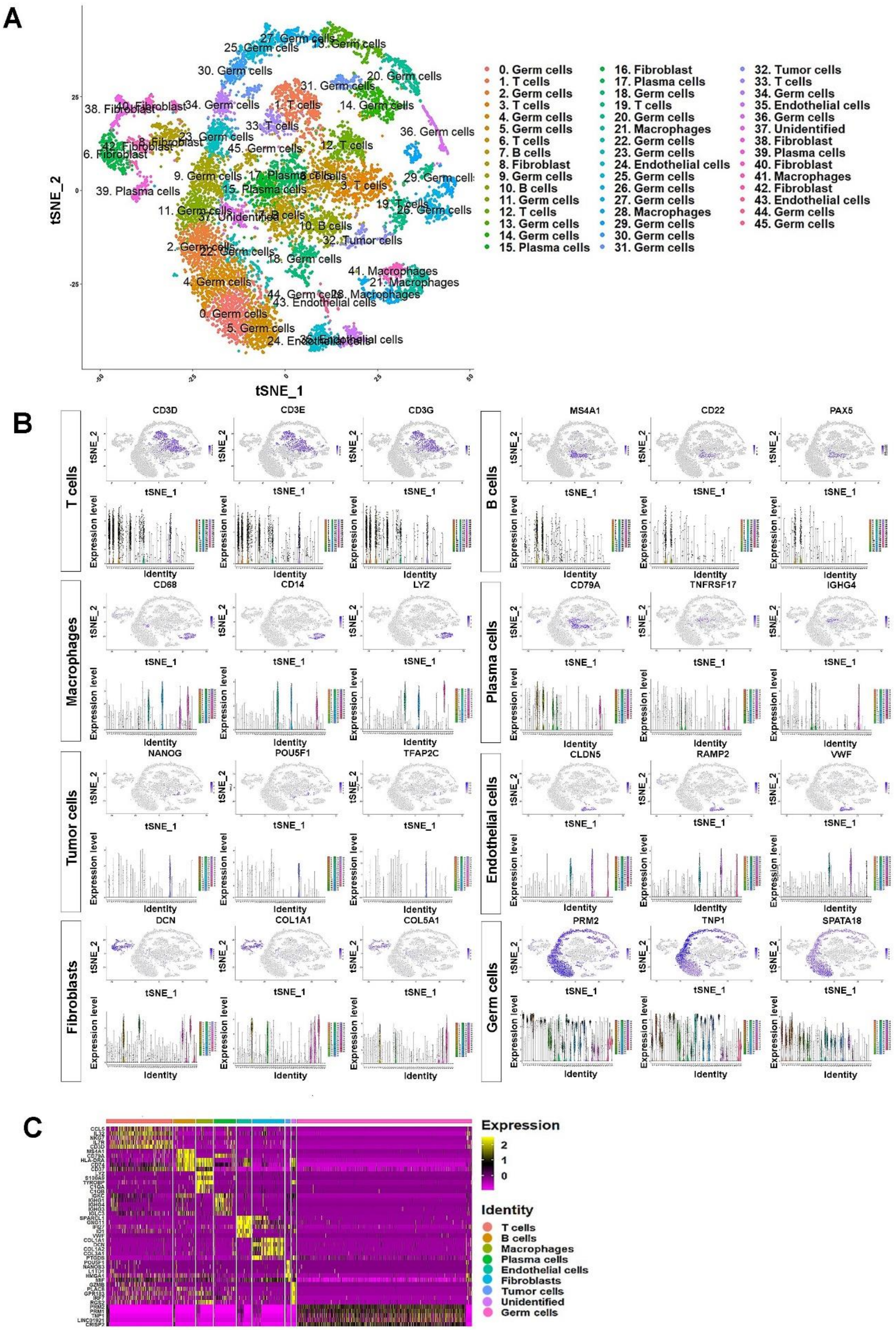
A
tSNE_2
tSNE_1
0. Germ cells
1. T cells
2. Germ cells
3. T cells
4. Germ cells
5. Germ cells
6. T cells
7. B cells
8. Fibroblast
9. Germ cells
10. B cells
11. Germ cells
12. T cells
13. Germ cells
14. Germ cells
15. Plasma cells
16. Fibroblast
17. Plasma cells
18. Germ cells
19. T cells
20. Germ cells
21. Macrophages
22. Germ cells
23. Germ cells
24. Endothelial cells
25. Germ cells
26. Germ cells
27. Germ cells
28. Macrophages
29. Germ cells
30. Germ cells
31. Germ cells
32. Tumor cells
33. T cells
34. Germ cells
35. Endothelial cells
36. Germ cells
37. Unidentified
38. Fibroblast
39. Plasma cells
40. Fibroblast
41. Macrophages
42. Fibroblast
43. Endothelial cells
44. Germ cells
45. Germ cells
B
T cells
CD3D
CD3E
CD3G
B cells
MS4A1
CD22
PAX5
Macrophages
CD68
CD14
LYZ
Plasma cells
CD79A
TNFRSF17
IGHG4
Tumor cells
NANOG
POU5F1
TFAP2C
Endothelial cells
CLDN5
RAMP2
VWF
Fibroblasts
DCN
COL1A1
COL5A1
Germ cells
PRM2
TNP1
SPATA18
Expression level
Identity
C
Expression
2
1
0
Identity
T cells
B cells
Macrophages
Plasma cells
Endothelial cells
Fibroblasts
Tumor cells
Unidentified
Germ cells

Supplemental Fig. 3: Clustering information of different cell landscape in normal testis and TGCT. A: tSNE presentation of major cell types and associated clusters in all samples. **B:** tSNE plots and Violin plots show the expression of selected markers for each cell type throughout 46 clusters. **C.** Heatmap of the top 5 significantly differentially expressed genes in each cell type analyzed.

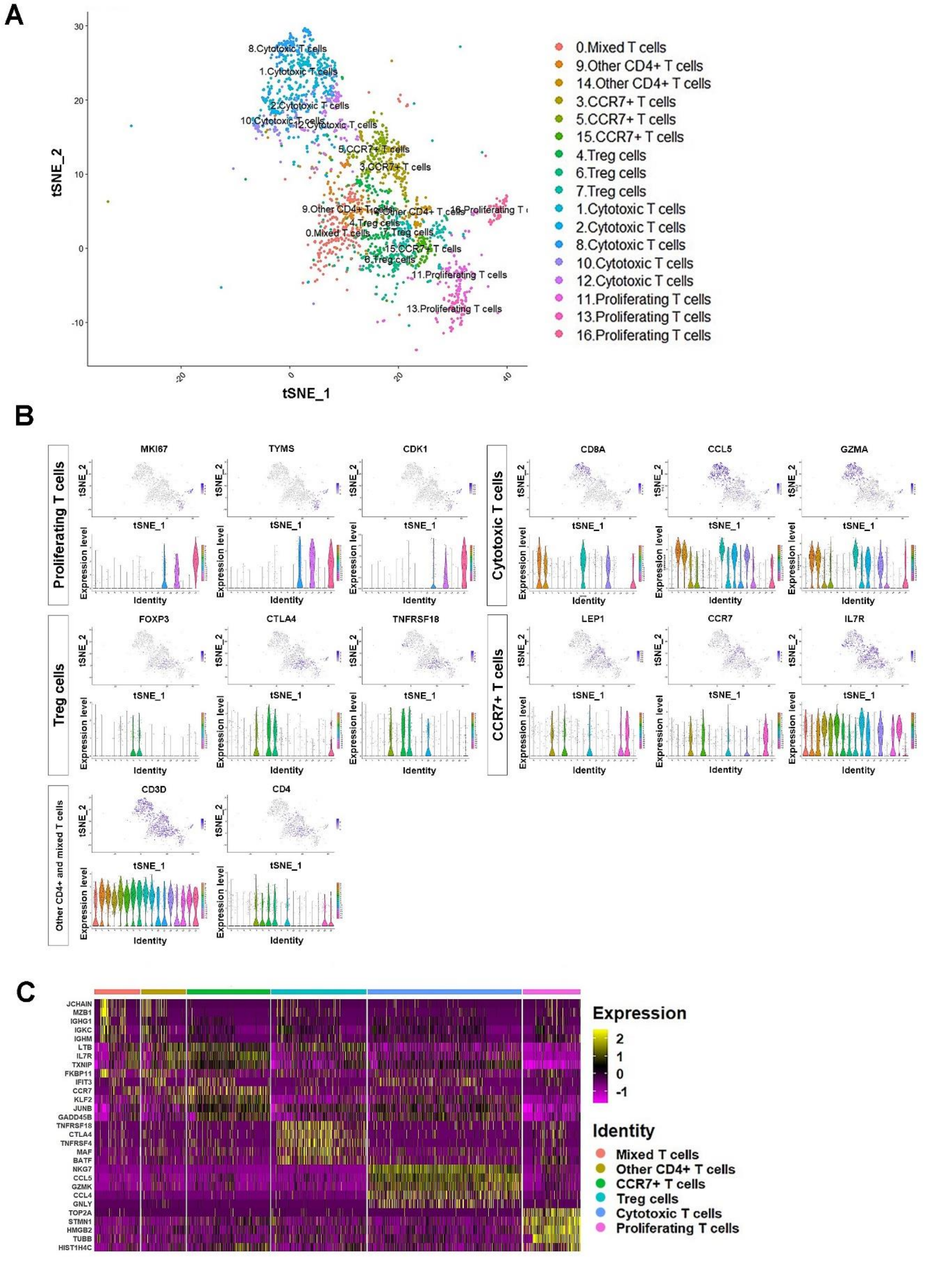
A
tSNE_2
tSNE_1
0.Mixed T cells
9.Other CD4+ T cells
14.Other CD4+ T cells
3.CCR7+ T cells
5.CCR7+ T cells
15.CCR7+ T cells
4.Treg cells
6.Treg cells
7.Treg cells
1.Cytotoxic T cells
2.Cytotoxic T cells
8.Cytotoxic T cells
10.Cytotoxic T cells
12.Cytotoxic T cells
11.Proliferating T cells
13.Proliferating T cells
16.Proliferating T cells
B
Proliferating T cells
MKI67
TYMS
CDK1
Cytotoxic T cells
CD8A
CCL5
GZMA
Treg cells
FOXP3
CTLA4
TNFRSF18
CCR7+ T cells
LEP1
CCR7
IL7R
Other CD4+ and mixed T cells
CD3D
CD4
Expression level
Identity
C
JCHAIN
MZB1
IGHG1
IGKC
IGHM
LTB
IL7R
TXNIP
FKBP11
IFIT3
CCR7
KLF2
JUNB
GADD45B
TNFRSF18
CTLA4
TNFRSF4
MAF
BATF
NKG7
CCL5
GZMK
CCL4
GNLY
TOP2A
STMN1
HMGB2
TUBB
HIST1H4C
Expression
Identity
Mixed T cells
Other CD4+ T cells
CCR7+ T cells
Treg cells
Cytotoxic T cells
Proliferating T cells

Supplemental Fig. 4: Clustering information of different T cell landscape in normal testis and TGCT. **A:** The tSNE plots align the T cell clusters. **B:** tSNE plots and Violin plots show the expression of selected marker for different T cell subtypes. **C:** Heatmap shows the top 5 differentially expressed genes by various T cell clusters in the studied samples.
